# Supplement study update for Reach Out: a multi-arm randomized trial of behavioral interventions for hypertension initiated in the emergency department: Reach Out Cognition

**DOI:** 10.1186/s13063-021-05806-4

**Published:** 2021-11-24

**Authors:** Lesli E. Skolarus, Mackenzie Dinh, Kelley M. Kidwell, Zahera Farhan, Candace Whitfield, Deborah A. Levine, William J. Meurer

**Affiliations:** 1 Department of Neurology, University of Michigan, Ann Arbor, USA; 2 Stroke Program, University of Michigan, Ann Arbor, USA; 3 Department of Emergency Medicine, University of Michigan, Ann Arbor, USA; 4 Department of Biostatistics, School of Public Health, University of Michigan, Ann Arbor, USA; 5 Department of Internal Medicine, University of Michigan Medical School, Ann Arbor, MI USA; 6 Institute for Healthcare Policy and Innovation, University of Michigan, Ann Arbor, USA; 7 Michigan Institute for Integrative Research in Critical Care (MCIRCC), University of Michigan, Ann Arbor, USA

**Keywords:** Hypertension, Emergency Medicine, Multiphase Optimization Strategy, Cognition, Health Equity

## Abstract

**Background:**

Reach Out is a factorial trial studying multicomponent behavioral interventions to reduce blood pressure in hypertensive emergency department patients. The original study protocol was published in June 2020. Here, we describe the updated protocol, including a supplemental study, Reach Out Cognition. Reach Out Cognition is a remote study that will assess the acceptability, feasibility, and satisfaction of digital, self-administered cognitive assessments and Bluetooth-enabled, self-measured blood pressure monitoring in the Reach Out population. We will also estimate the prevalence of mild cognitive impairment in Reach Out participants.

**Methods:**

Reach Out Cognition includes remote enrollment and follow-up assessments. Reach Out Cognition extends Reach Out data collection past the current 12 months to 15 and 18 months. Participants will be Reach Out participants who complete their 12-month outcome assessments and opt to continue in the cohort study. Participants will continue to receive the Reach Out intervention, consisting of (1) daily healthy behavior text messaging and (2) weekly self-measured blood pressure monitoring. Blood pressure will be measured using Bluetooth-enabled self-measured blood pressure monitoring devices, and cognition will be measured using digital self-administered cognitive assessments at 12, 15, and 18 months.

**Discussion:**

Reach Out Cognition will explore the potential of remote, digital, self-administered assessments of blood pressure and cognition among predominantly working-age Black Americans. Reach Out Cognition will inform future clinical trials and clinical remote monitoring of blood pressure and cognition that may lead to new approaches to treating and reducing hypertension and cognitive disparities.

**Trial registration:**

ClinicalTrials.gov NCT03422718. The record was first available to the public on January 30, 2018, prior to the enrollment of patients on March 25, 2019.

## Administrative information

**Table:** 

Title	Supplement study update for Reach Out: a multi-arm randomized trial of behavioral interventions for hypertension initiated in the Emergency Department: Reach Out Cognition
Trial registration	ClinicalTrials.gov Identifier: NCT03422718
Protocol version	Version 13.0; October 8, 2020
Funding	Funded by National Institutes of Health, National Institutes of Minority Health and Disparities R01 MD011516- 04S1
Author details	Lesli E. Skolarus^1,2^, Mackenzie Dinh^3^; Kelley M. Kidwell^4^; Zahera Farhan^3^; Candace Whitfield^3^; Deborah A. Levine^1,2,5,7^, William J. Meurer^1,2,3,6,7^ 1. Department of Neurology, University of Michigan 2. Stroke Program, University of Michigan 3. Department of Emergency Medicine, University of Michigan 4. Department of Biostatistics, School of Public Health, University of Michigan 5. Department of Internal Medicine, University of Michigan Medical School, MI, USA. 6. Institute for Healthcare Policy and Innovation, University of Michigan, Ann Arbor, USA 7. Michigan Institute for Integrative Research in Critical Care (MCIRCC), University of Michigan, Ann Arbor,USA
Name and contact information for the trial sponsor	National Institute on Minority Health and Health Disparities (NIMHD) National Institutes of Health 6707 Democracy Boulevard, Suite 800 Bethesda, MD 20892-5465 Telephone: 301-402-1366 Fax: 301-480-4049 Email: NIMHDinfo@NIMHD.NIH.gov
Role of sponsor	The study sponsor and funders provided peer review of the study design. The sponsor had/will have no role in the collection, management, analysis, and interpretation of data; writing of the report; or the decision to submit the report for publication. They will not have ultimate authority over any of these activities.

## Introduction

### Background

This paper outlines the Reach Out Cognition study protocol. The current study is a supplemental study that focuses on participants who have completed their 12-month follow-up in the Reach Out clinical trial. The original detailed protocol for Reach Out has been previously published [1]. Reach Out is a factorial trial studying multicomponent behavioral interventions to reduce blood pressure (BP) in the emergency department (ED) patient population. The primary objective was to determine which behavioral intervention components or “dose” of the components contributes to a reduction in systolic BP at 1 year. The secondary objective was to determine the effect of primary care provider (PCP) appointment scheduling and transportation on primary care follow-up of hypertensive participants initiated from an urban, safety net ED. Since March 2019, 849 participants have been enrolled from the ED, most working-age Black Americans. The final participant was recruited in March 2020, with the last follow-up visit completed in April 2021.

Large epidemiological studies have shown an association between hypertension and incident mild cognitive impairment (MCI) and dementia [2, 3]. These associations are particularly noted for midlife hypertension and dementia [4, 5]. Black Americans also have the highest incidence and prevalence of hypertension and the highest incidence of dementia of any US race/ethnic group [6]. Black Americans’ higher cumulative BP levels may contribute to their greater risk of later-life cognitive decline compared to White Americans [7]. Treatment of hypertension may reduce the incidence of MCI and dementia [8]. Thus, innovative solutions to hypertension treatment in Black Americans are needed.

### Significance

In this context, we will implement Reach Out Cognition, extending Reach Out data collection past the current 12 months to 15 and 18 months. Reach Out Cognition is a remote study that includes remote enrollment and follow-up assessments. During Reach Out Cognition, we will assess the acceptability, feasibility, and satisfaction with remote, digital, self-administered cognitive assessments (aim 1) and Bluetooth-enabled self-measured BP monitoring (aim 2). During Reach Out, participants self-measured BP using study-provided devices and texted their BP readings to the research team. Blue-tooth-enabled BP monitors may be more advantageous because they allow the transfer of BP data to a smartphone app eliminating reporting bias and misreporting [9]. In addition, cognitive assessments were not performed in Reach Out and thus will be added to Reach Out Cognition. In the final aim, we will estimate the prevalence of MCI in Reach Out participants. Importantly, Reach Out Cognition will be implemented in a predominantly working-age Black population recruited from a safety net ED in Flint, Michigan.

## Methods

### Participants

**Fig. 1:**
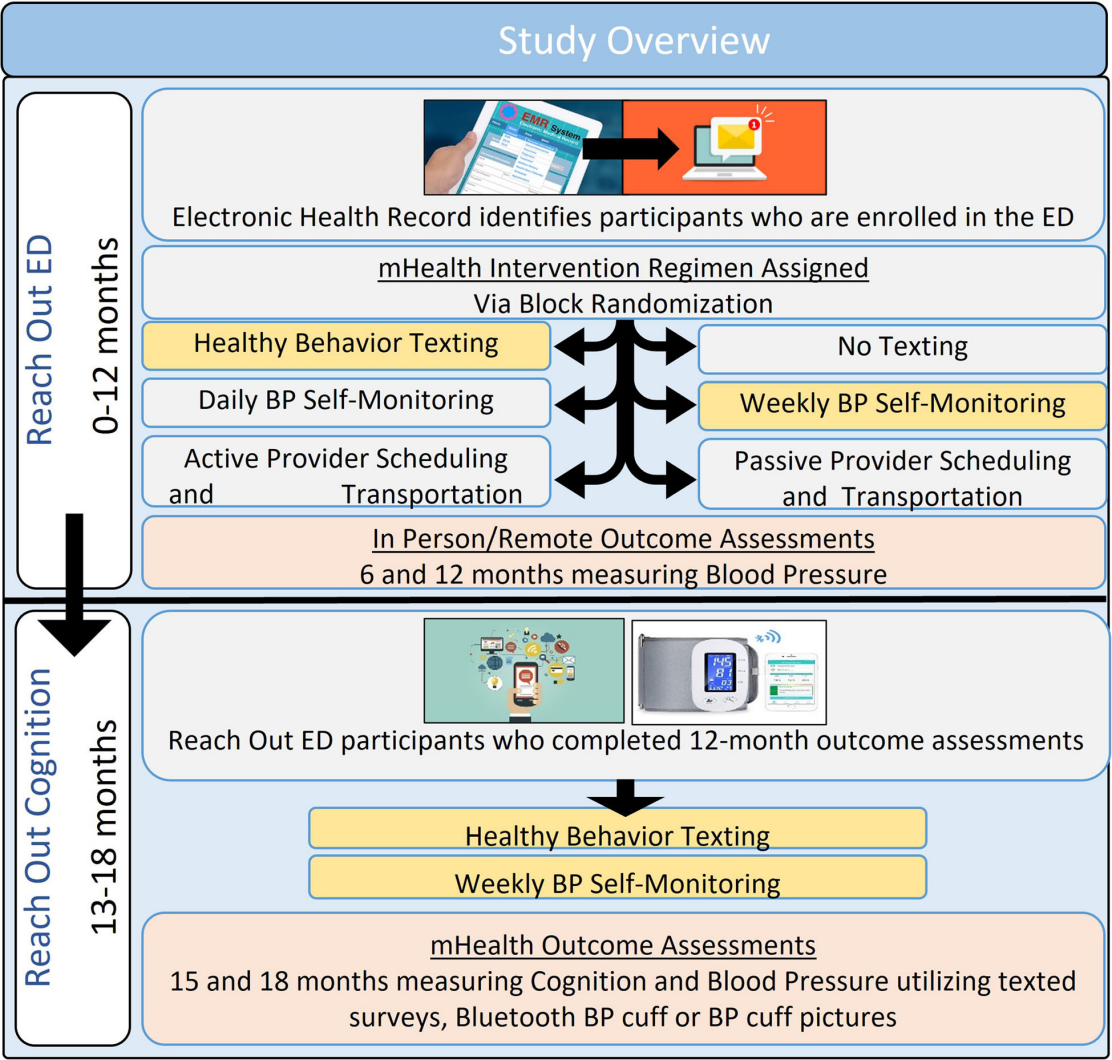
Study overview

Adults are eligible for Reach Out Cognition if they were previous participants of Reach Out who were no more than 6 months from completion of their 12-month outcome assessment and had normal cognition (Fig. 1). A total of 212 people completed their Reach Out 12-month outcomes and will be approached for Reach Out Cognition.

#### Participants and cognition

Participants with dementia were excluded from Reach Out. Dementia was defined as a clinical diagnosis, the inability to stay home alone for 24 h, or the inability to provide consent. In Reach Out Cognition, we will screen for MCI. Prior to consent for Reach Out Cognition, we will administer the telephone-Montreal Cognitive Assessment (T-MoCA-Short) to Reach Out participants [10]. The T-MoCA-Short measures verbal fluency, recall, and orientation for a total of 12 points. The T-MoCA-Short has high accuracy in detecting MCI, including vascular cognitive impairment, in Black and White individuals and is recommended by the NINDS-CSN Harmonization Standards Working Group [11, 12]. We used a cut-off score < 8 (sensitivity = 42%, specificity = 88%, positive predictive value = 69%) [10] to identify Reach Out participants with MCI. The T-MoCA-Short scores will be used to estimate the prevalence of MCI in Reach Out participants. Participants who are found to have MCI will be excluded from Reach Out Cognition because we are exploring the implementation of self-measured BP monitoring and detection of transition to MCI in participants with normal baseline cognition.

### Study procedures

**Fig. 2:**
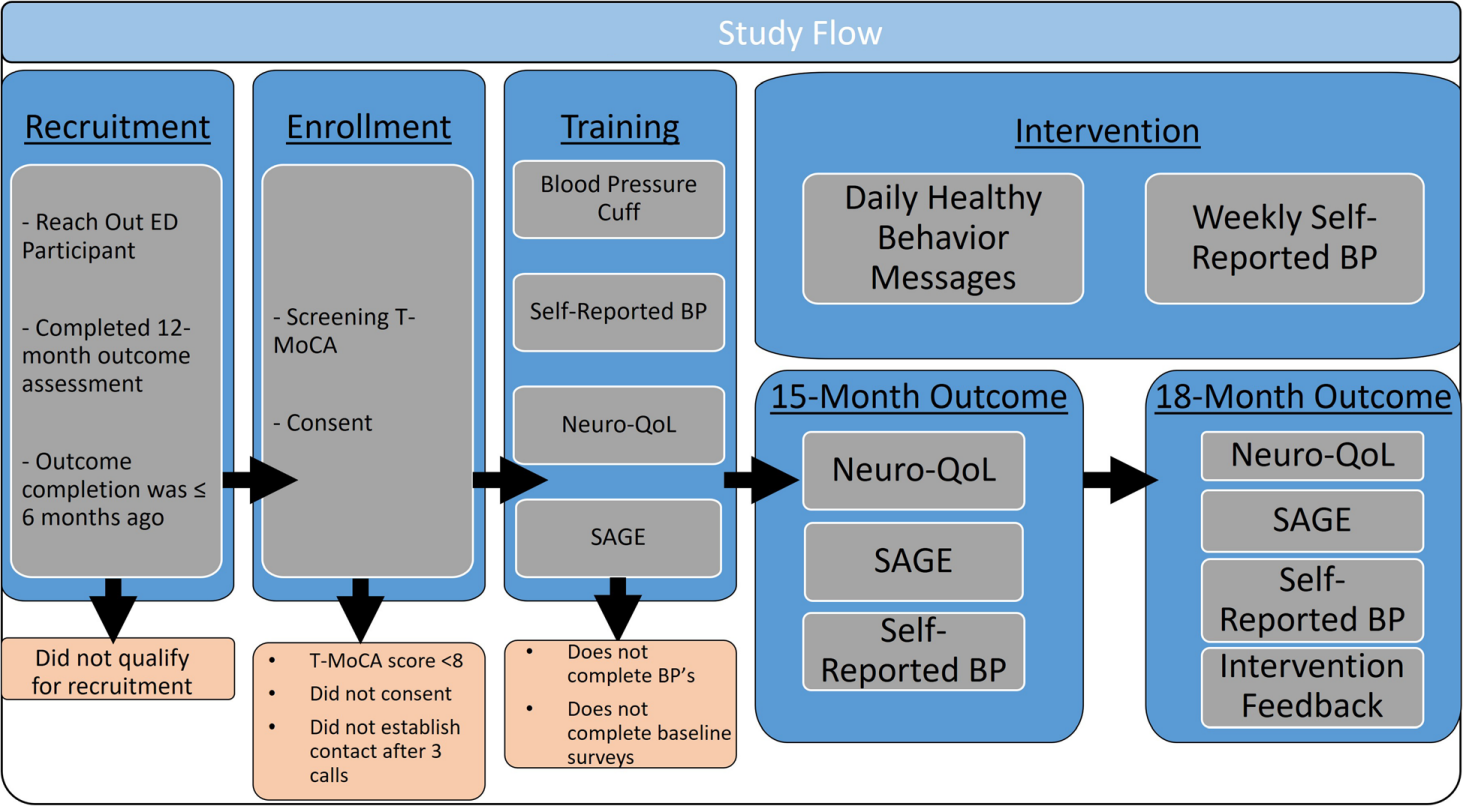
Study flow

There will be three episodes of contact between the research team and participants (recruitment, enrollment, and training) before the initiation of the intervention (Fig. 2). All aspects of recruitment, enrollment, and training are conducted remotely via phone, video conferencing, written materials, or online training (reachouted.com). First, eligible Reach Out participants will be invited to participate in Reach Out Cognition. For those participants who are interested, a research assistant will assess the participant’s cognitive ability, utilizing the T-MoCA-Short. If the participant is found to have normal cognition, the research assistant will then proceed to consent. All participants enrolled in the study will be mailed a packet that includes a wireless, Bluetooth-enabled OMRON 7 series upper arm BP monitor (BP7350) or, for those who need a larger cuff, an OMRON 7 series wrist BP monitor (BP6350). The packet also includes participant instructions for taking their BP, study-specific instructions for texting their BP, instructions for completing outcomes based on their phone’s capability, an individualized MoCA feedback form, and a checklist of enrollment elements.

### Training and assessments

#### BP

Of note, Reach Out was based on text messages and thus enrolled participants with feature phones or smartphones. Our goal is inclusivity and generalizability; thus, Reach Out Cognition included participants with either phone type, albeit procedures differ (Table 1).

**Table 1:** Study assessment schedule

Baseline (12-month)	15-month	18-month
Self-reported BP - Feature phone o Picture/texts - Smartphone o Texted/emailed report	Self-reported BP - Feature phone o Picture/texts - Smartphone o Texted/emailed report	Self-reported BP - Feature phone o Picture/texts - Smartphone o Texted/emailed report
T-MoCA (baseline status) - Feature phone o Telephone - Smartphone o Telephone	Sage - Feature phone/smartphone o Texted survey or paper by request	Sage - Feature phone/smartphone o Texted survey or paper by request
Sage - Feature phone/smartphone o Texted survey or paper by request	Neuro-QoL - Feature phone/smartphone o Texted survey or paper by request	Neuro-QoL - Feature phone/smartphone o Texted survey or paper by request
Neuro-QoL - Feature phone/smartphone o Texted survey or paper by request		Intervention feedback - Feature phones/smartphones o Texted survey or paper by request o Telephone

Participants with smartphone capability will be texted instructions to download the OMRON App associated with the Bluetooth-compatible BP monitoring device, download their data from the app, and text/email data to the study team. Participants with feature phones (no app capability) will be trained on how to use the BP digital monitoring device with up to 120 BPs readings stored on the device, how to photograph the process of obtaining BP measurements and submit the photograph via text to the study team. While ideally, the texted picture would include the BP measurement and the BP cuff on the participant, we only require confirmation that the BP cuff is on the participant’s arm as a measure of the validity of the measurement.

#### Cognitive

Self-administered cognitive measures will be assessed using two validated measures: (1) Self-Administered Gerocognitive Exam (SAGE) and (2) Neuro-QoL-Cognition. The SAGE is a 22-point self-administered assessment (higher scores indicate better cognitive performance). It includes the cognitive domains of orientation (date, 4 points), language (picture naming 2 points and verbal fluency 2 points), memory (2 points), executive function (modified Trails B 2 points and problem-solving task 2 points), calculations (2 points), abstraction (2 points), and visuospatial abilities (copying 3-dimensional constructions 2 points and clock draw 2 points). It has been validated and found to have a sensitivity of 79% of detecting any cognitive impairment and a specificity of 95% [13]. SAGE has been completed in the community setting by over 1000 participants, of which 8% were African American, and is available as an app [14]. However, the SAGE app, BrainTest, is only available on a tablet, which is not universally accessible to our participants [15]. The SAGE will be administered through a Qualtrics survey using features such as text boxes for the cognitive domains of orientation, language, abstraction, and calculation constructs, freestyle drawing for visuospatial construct questions, and heat mapping for executive function construct questions. In addition to measures of cognition, participant perceptions of cognition are also important. Thus, we will also include Quality of Life in Neurological Disorders (Neuro-QoL) Cognitive Function short form (v2.0). Neuro-QoL, initiated by NINDS, is a validated measure of quality of life, including cognition [16]. The Neuro-QoL Cognitive Function Short Form v2.0 includes (1) general concerns, which includes perceived difficulties with everyday cognitive tasks such as memory, language, attention, and decision making, and (2) executive function, which queries perception in planning, organizing, calculating, and learning [16]. It was also administered through a Qualtrics survey. In addition, the SAGE and Neuro-QoL were also available as paper-based assessments with the option to mail to participants with feature phones.

#### Training procedures

If participants do not complete their BP and cognitive assessments after the texted instructions, participants will be called and texted once per week for 1 month for assistance (maximum of four follow-up texts and four follow-up calls). Participant trainings with the study staff offer the possibility to address any questions that may have occurred during the initial enrollment into the study, assistance with downloading the OMRON app, and assistance in completing the baseline digital cognitive measures and self-reported BP. Participants will also be texted a link to the Reach Out website to offer visual examples and training for completing enrollment elements. Each week participants will be texted a personalized message with instructions specifying what they may still have to complete for enrollment. If participants do not respond and do not complete training for 1 month post-enrollment, they will be considered “unable to train” and withdrawn from the intervention.

### Reach out intervention

Participants will receive the Reach Out intervention at moderate intensity, receiving daily healthy behavior text messages and weekly prompted self-measured BP monitoring reminders. As described in Reach Out, the healthy behavior text messages are tailored and targeted, based on social cognitive theory, addressing essential lifestyle interventions to reduce BP such as salt reduction, increase in fruit and vegetable intake, and increase in physical activity. Tailored text messages are based on whether the participant took an antihypertensive medication, had a PCP, and medicationadherence level. Weekly audit and feedback messages are tailored based on whether the participant’s self-reported BP was lower or higher than the prior week.

### Study engagement strategies

All participants will be mailed a Bluetooth BP cuff at enrollment (about $50 value). Participants will be given $20 for completing the 15-month outcome assessment and $30 for completing the 18-month outcome assessment. Participants will also be sent a newsletter including general study updates, outcome information, and a study staff section highlighting a particular member of the research team. All the shipped materials include means to contact Reach Out with questions regarding intervention. Additional items, including a pen and magnet, will also be sent with the study logo to increase study visibility.

### Assessments

Outcome assessments will be based on whether the participant has a smartphone or feature phone (Table 1). Participants with smartphones will be asked to email/text their BP data downloaded from their OMRON app. Participants with feature phones will be asked to include a photograph of themselves taking their BP when they text in their BP. Cognitive assessments will be conducted through text message linked online surveys.

Assessments will be offered in a flexible schedule and format to best accommodate participants. The assessments will be conducted over 6 weeks to allow participants ample time to complete them. Participants will be contacted at least five times, with three text messages and two calls throughout the daytime, evening, and weekend hours to encourage assessment completion. If the participant has difficulty with self-measured cognitive and BP assessments, the study team will conduct a remote visit to provide assistance or collect BP measurements over the phone.

We will measure acceptability, feasibility, and satisfaction, which are necessary for large-scale use of digital BP and cognition measures [17]. We will also estimate the prevalence of MCI in the Reach Out population.

Acceptability is defined as the participant’s perception that digital assessments are achievable and agreeable. We will measure acceptability as a form of engagement which will be assessed by the proportion of Reach Out participants who enroll in Reach Out Cognition. In addition, at the end of the study, we will retrospectively measure acceptability using a validated 4-item, written at a 5th-grade reading level [17, 18]. We will administer separate scales to assess the acceptability of self-reported blood pressure monitoring with the app and digital cognitive assessments.

Feasibility is the extent to which digital assessments can be successfully carried out. Feasibility will be assessed by the proportion of participants who complete the digital cognitive assessment and the BP assessments separately. We will declare feasibility if greater than 50% of participants provide at least one cognitive assessment.

Satisfaction with self-measured BP monitoring using an app and self-administered and digital cognitive assessments will be measured with a text survey to all participants. Satisfaction is also queried through questions of overall satisfaction with Reach Out Cognition and training provided. In addition, participants will also be asked for feedback on interactions with the study team, technology barriers, and overall experiences through Reach Out Cognition. Participant’s experiences of COVID-19 will also be measured, including how COVID-19 may have impacted the ability to receive healthcare and self-monitor blood pressure.

### Sample size

The sample size is dependent on the number of Reach Out participants who have completed a 12-month follow-up visit.

### Statistical methods

Descriptive statistics will be used to evaluate participant acceptability, feasibility, and satisfaction. The denominator for the acceptability outcome is the number of Reach Out participants who complete the 12-month assessments. We will also determine the acceptability scores for self-reported blood pressure monitoring with the app and digital cognitive assessments (4–20). We will separately determine the feasibility by digital measure (i.e., BP vs. Cognition), phone type (i.e., smartphone vs. feature phone), and operating system type (iOS vs. Android vs. Windows) as a continuous and dichotomous (> 50% completion of each assessment type) measure. Satisfaction will be assessed for all participants. Given the difference in procedures by phone type, we will compare satisfaction between the smartphone and feature phone users using a Kruskal-Wallis test.

We anticipate that approximately 10–20% of Reach Out participants will have previously undiagnosed MCI identified by the T-MoCA-short. We will not test a formal hypothesis; however, we will calculate the 95% confidence interval for the prevalence of MCI and examine the interval to see if it excludes 20% (if the proportion is less than 20%) or 10% (if the proportion is greater than 20%). In addition, the Neuro-QOL cognition instrument provides age normalized values and standard errors with a population mean T-score of 50 and standard deviation of 10; we will determine how much the Reach Out Cognition cohort differs from the expected values by graphically presenting the T-scores from the Reach Out Cognition participants [16]. Using existing data on the trajectory of change in T-MoCA-short based on age which showed that participants had an annual decline of 1–2 points per year on the T-MoCA-short [19], we will develop estimates of the likely cognitive status of Reach Out participants who screened positive for MCI during Reach Out Cognition enrolment. Assuming the 12 month T-MoCA-short for each patient represents (i) a 1 point annual change and (ii) a 2 point annual change, we will separately present how many Reach Out participants would have had MCI 12 months prior.

## Discussion

Reach Out Cognition is a remote study assessing the acceptability, feasibility, and satisfaction of digital measures of cognition and BP in a racially diverse population of hypertensive, predominately working age, ED patients of an urban safety-net hospital. Studies suggest BP lowering is a promising intervention to reduce late-life cognitive decline, MCI, and dementia [7, 8]. Studies of BP lowering and cognition in younger, racially diverse populations are needed, yet they are limited by data collection challenges using in-person study visits. Digital measures of cognition reflect an emerging area of healthcare, research, and commercial business. Reach Out Cognition will explore the integration of behavioral approaches to BP reduction, such as self-monitoring and feedback, with novel Bluetooth BP cuff integration and self-administered remote digital cognitive surveys.

### Strategy and pitfalls

This work has several limitations. Our results only apply to participants without dementia initially recruited from a safety-net ED and anticipated to be discharged home. Within Reach Out Cognition, MCI is defined as a T-MoCA-Short score of < 8, although other thresholds could have been chosen based on a trade-off of sensitivity and specificity [10]. We are limited to online assessments of cognition, rather than app-based because we enroll participants with and without smartphone capabilities. Additionally, we administered the SAGE online, which is a modification from the standard paper-based assessment. At the start of the trial, there was a lack of validated digital self-administered cognitive assessments [20]. However, projects to develop these tools, such as the Ambulatory Methods for Measuring Cognitive Change (M2C2), are underway [21]. The Neuro-QoL is validated, and there is some precedence for online administration [16, 22].

Our use of Bluetooth BP cuff technology also has limitations. Ideally, the BPs could be accessed from the app by the research team. However, in Reach Out Cognition, the BPs must be manually shared through email or screenshot of the app, which will then be texted to the research team. Future studies should consider obtaining access to the BP application programming interface (API), which will allow participant data to flow from their device to the research team, given appropriate participant permission [23]. Finally, Bluetooth technology may become un-synced between BP monitors and phones. We conduct frequent technology check-ins with participants to ensure there is support if needed to address potential issues.

In summary, Reach Out Cognition will inform future clinical trials and clinical remote monitoring of blood pressure and cognition that may lead to new approaches to treating and reducing hypertension and cognitive disparities.

## Trial status

Recruitment for Reach Out Cognition started on September 24, 2020. As of June 2, 2021, we had enrolled 93 participants. The approximate date of outcome completion is February 2022.

## Data Availability

As noted above, individual patient level data will be available through the University of Michigan institutional archive after de-identification.

## Abbreviations

API: Application programming interface
BP: Blood pressure
ED: Emergency department
M2M: Ambulatory Methods for Measuring Cognitive Change
Neuro-QoL: Quality of Life in Neurological Disorders
NIMHD: National Institute on Minority Health and Disparities
NINDS*-*CSN: The National Institute of Neurological Disease and Stroke-Canadian Stroke Network
PCP: Primary care provider
SAGE: Self-Administered Gerocognitive Exam
T-MoCA: Telephone-Montreal Cognitive Assessment-Short
